# Association between nutrition self-efficacy, health locus of control and food choice motives in consumers in nine European countries

**DOI:** 10.1177/13591053241249863

**Published:** 2024-05-14

**Authors:** Barbara J Stewart-Knox, Rui Poínhos, Arnout RH Fischer, Audrey Rankin, Brendan P Bunting, Bruno MPM Oliveira, Lynn J Frewer

**Affiliations:** 1 University of Bradford, UK; 2 University of Porto, Portugal; 3 Wageningen University, Netherlands; 4 Independent researcher, UK; 5 Ulster University, UK; 6 Newcastle University, UK

**Keywords:** food choice motives, food choice questionnaire, health locus of control, nutrition self-efficacy, survey

## Abstract

We investigated associations between food choice motives and psychological determinants of dietary health behaviour change (nutrition self-efficacy, NS-E, and health locus of control, HLoC) among 9381 participants (18–65 years, 49.4% females) from nine European countries. Price was the highest rated food choice motive. Higher importance of all motives was associated with higher NS-E and with higher Internal HLoC. Relationships between food choice motives and External HLoC were also in the expected direction in showing negative associations with Health, Natural Content, Weight Control, Mood and Sensory Appeal. Higher External HLoC was also associated with perceived greater importance of ‘external’ motives Ethical Concern, Familiarity and Convenience. Relationships between External HLoC and food choice motives were not all in the expected direction. Price was unrelated to External HLoC. Females rated the importance of all motives higher than males. People with less education ascribed greater importance to Price in motivating food choices. Together, these findings imply that self-efficacy and health locus of control should be considered along with motivations for food choice in dietary health promotion.

## Introduction

The promotion of healthy eating in the endeavour to reduce incidence of non-communicable disease remains a challenge for public health in Europe (European Environment Agency, 2022). This may in part be caused by ineffective interventions targeting food choice and individual differences in consumer psychology (Reinders et al., 2023). In order to design more targeted intervention to encourage healthier eating, this analysis has considered determinants of food choice at the individual level that are concerned with motivation, along with psychological factors that hold potential to inform intervention to enhance self-belief, self-efficacy and perceived control (Greiner et al., 2018; Reinders et al., 2023).

Motives underlying food choices are shaped by a wide range of individual, social and environmental factors (Rankin et al., 2018), are context dependent (Alles et al., 2017), changeable over time (Marty et al., 2021) and as such, are modifiable and should be amenable to change through intervention. Food choice motives have been shown to be good predictors of nutrient intake (Daly et al., 2023) and dietary intake of fruit and vegetables (Konttinen et al., 2013; Miyahira et al., 2023), rice (Castanho et al., 2023) and sustainable food (Alles et al., 2017), as well as other food-related behaviour such as intention to reduce food waste (Pandey et al., 2023) and to adopt personalised nutrition (Rankin et al., 2018). Food choice motives are also associated with Body Mass Index (BMI) (Daly et al., 2023; da Silva et al., 2022). It has also been established that food choice motives vary between societal groups (Daly et al., 2023; da Silva et al., 2022; Markovina et al., 2015). People with higher socio-economic status (SES) are more likely to perceive health as an important motive for making food choices, while those of lower SES more often perceive price and familiarity as more important (Konttinen et al., 2013). Food choice motives also appear to vary cross-nationally and between genders (Pearcey and Zhan, 2018). These factors should therefore be considered in the analyses of socio-demographic factors when understanding motives for food choice. Psychological personality traits may also be related to food choice motives (Berezowska et al., 2017; Keller and Siegrist, 2015). This analysis considers two such traits, Health locus of control and Self-efficacy, both of which are facets of social cognitive theory associated with behaviour change (Bandura, 1997) and which have been shown to be relevant to healthy food choice (Stewart-Knox et al., 2021).

Health locus of control (HLoC) is a psychological construct that refers to the extent to which individuals believe they are in control of their health (Wallston et al., 1978). Internal HLoC is the degree to which health is perceived to be influenced by one’s own actions, while external HloC is the degree to which health is viewed as the result of the actions of others or governed by chance (Rotter, 1966; Wallston et al., 1978). HLoC can be related to food choice. Higher internal HLoC has been associated with healthier food choices (Cheng et al., 2016; Cobb-Clark et al., 2014; Grotz et al., 2011), willingness to buy organic food (Lee et al., 2019) and purchase of locally produced food (Hempel and Roosen, 2022). There is also some evidence to suggest that higher internal HLoC is associated with greater perceived importance of health over taste or convenience in motivating food choices (Cohen and Azaiza, 2007). External HLoC appears to have been less researched than internal HLoC. Higher external HLoC tends to be associated with less healthy eating (Bennett et al., 1994; Grotz et al., 2011; Omidvar et al., 2003), and being overweight (Gruszka et al., 2022).

Although HLoC is considered a trait (Ryon and Gleason, 2014), and therefore enduring in the individual, it appears to vary by gender and between different social groups. Previous research has consistently found internal HLoC to be higher in females (Paxton and Sculthorpe, 1999; Poortinga et al., 2008; Pudrovska, 2015), and in those of higher SES (Jang and Baek, 2018; Poortinga et al., 2008). External HLoC, in contrast, appears to be higher in males (Grotz et al., 2011; Poortinga et al., 2008; Pudrovska, 2015) and those of lower SES (Grotz et al., 2011; Poortinga et al., 2008). Nevertheless, there are indications that HLoC can change for individuals, as it tends to be higher among older age groups (Poortinga et al., 2008). There is even some limited evidence that HLoC can be amenable to intervention (Ryon and Gleason, 2014). The current analysis seeks to establish how food choice motives are related to HLoC to inform potential interventions that tap into internal HLoC and counter external HLoC in the endeavour to motivate healthy, sustainable food choices.

Self-efficacy is the degree to which an individual feels able to achieve a particular goal (Bandura, 1997), and Nutrition Self-efficacy (NS-E) is considered a good predicter of nutrition-related behaviour (AbuSabha and Achterberg, 1997). Greater self-efficacy has been consistently linked to healthier food choices (Anderson et al., 2007; Davison et al., 2015; Greiner et al., 2018; Lo et al., 2019; Naughton et al., 2015a; Smith et al., 2020; Swan et al., 2015; Williams et al., 2012) and with greater fruit and vegetable intake (Anderson et al., 2007; Appleton and Adams, 2023; Greiner et al., 2018; Kehm et al., 2017; Kushida et al., 2017; Liou and Kulik, 2020; Lo et al., 2019; Smith et al., 2020). People with higher self-efficacy engage in less snacking behaviour (Churchill et al., 2019), are more likely to read labels on food (Christoph et al., 2016), choose locally produced foods (Jung et al., 2023) and intend to reduce food waste (Pandey et al., 2023). Previous studies have established that higher self-efficacy is required to lose weight through dieting (Annesi, 2015a, 2015b, 2011; Freedman and Rubinstein, 2010; Paxton and Sculthorpe, 1999). Higher self-efficacy has also been associated with stronger response to healthy eating intervention (Annesi, 2018; Partridge et al., 2017; Stewart-Knox et al., 2021).

Evidence for gender differences in nutrition self-efficacy is mixed (Paxton and Sculthorpe, 1999; Pudrovska, 2015) and there does not appear to be any evidence linking nutrition self-efficacy to age or education level, which is in keeping with the assumption that self-efficacy is a fairly stable trait (Bandura, 1977). That self-efficacy tends to be higher in those of higher SES (Paxton and Sculthorpe, 1999), however, could imply some environmental influence. There is also evidence to imply that self-efficacy may be amenable to intervention (Guillaumie et al., 2012; Jamshidi et al., 2023; Kehm et al., 2017; Newby et al., 2020; Partridge et al., 2017; Roach et al., 2003; Shi et al., 2018; Smith et al., 2020). Meta-analysis of available empirical data (Newby et al., 2020), however, concluded that any change in self-efficacy in response to intervention tended to be small. This analysis, therefore, assumes that NS-E is a fairly stable trait which may be modifiable to some degree through intervention. In understanding how food choice motives relate to NS-E, it should be possible to target interventions to motivate healthy, sustainable eating more effectively.

Relatively few healthy eating initiatives appear to have taken psychological factors into account when tailoring intervention or assessing efficacy and behaviour change (Reinders et al., 2023). It is recommended that self-efficacy and HLoC be considered together in understanding health behaviour (AbuSabha and Achterberg, 1997), yet little is known about how self-efficacy and HLoC operate in motivating food choice (Reinders et al., 2023). HLoC and NS-E are relatively stable traits that may need to be taken into account in the design of interventions to modify food choice motives. The aim of this secondary analysis, therefore, has been to inform ways to tailor health promotion content to individual food choice motives given their association with health control beliefs and self-efficacy, whilst controlling for gender, age, education level and country. From the literature it was predicted that high self-efficacy would be associated with motives related to health (Davison et al., 2015; Ferranti et al., 2014; Greiner et al., 2018; Lo et al., 2019; Naughton et al., 2015a; Smith et al., 2020; Swan et al., 2015; Williams et al., 2012) and body weight control (Annesi, 2015a, 2015b, 2011; Freedman and Rubinstein, 2010; Paxton and Sculthorpe, 1999). It was also predicted that higher internal HLoC would be positively related to food choice motives associated with health and weight control (Cheng et al., 2016; Cobb-Clark et al., 2014; Cohen and Azaiza, 2007; Grotz et al., 2011), natural content (Lee et al., 2019) and ethical concern (Hempel and Roosen, 2022). External HLoC, which is associated with less healthy eating (Bennett et al., 1994; Grotz et al., 2011; Gruszka et al., 2022; Omidvar et al., 2003), was expected to be related to motives more influenced by external factors such as price and convenience.

## Methods

Ethical approval for the survey was granted by the Newcastle University Faculty of Science, Agriculture and Engineering ethics committee and all procedures for data collection were in accordance with the 1964 Helsinki Declaration and its later amendments. The survey questionnaire was designed and piloted by the authors. Nine countries were selected for survey with the aim of covering Northern (Norway), Southern (Spain, Portugal), Western (Ireland; UK), Eastern (Poland; Greece) and central (Germany; Netherlands) European regions. Given the extent of sampling required and the need for representativeness, a social research company (GfK) with capability across Europe was employed to recruit respondents and administer the survey. Volunteers were drawn from the existing GfK panel and quota sampled to be representative of each county in terms of gender, age and education level. Additional research agencies were subcontracted by GfK to supplement panels were required, such as in Ireland to achieve the required age range. A total of 29,450 people were initially contacted of whom 31.9% responded. Data were collected on-line in the Netherlands, United Kingdom (UK), Ireland, Germany, Portugal, Spain, Greece, Poland and Norway during February and March 2013 as part of the Food4Me survey on personalised nutrition.

### Materials

Questionnaire content was informed by prior qualitative research and drawing on psychological theory of behaviour change to increase health (Rankin et al., 2017; Stewart-Knox et al., 2013). The questionnaire was initially developed in English, then translated into the respective language of each country by each partner centre and then back-translated into English. Demographic data were collected (gender, age and education level). Education level was aligned to the International Standard Classification of Education (ISCED) system and then classified into one of three groups (level 0–2 = low; level 3–4 = middle; level 5–6 = high). Raw data and survey materials in all languages can be accessed at https://doi.org/10.5281/zenodo.7896317.

#### Food choice motives

The food choice questionnaire (FCQ) (Steptoe et al., 1995) identifies food choice motives on nine dimensions and has been shown to be valid for use in different countries (Markovina et al., 2015; Pearcey and Zhan, 2018). The FCQ comprised 36 items preceded by the statement ‘It is important to me that the food I eat on a typical day’. Although the FCQ was originally validated as a 4-point scale (Steptoe et al., 1995), it usually employed as a 5 or 7 point scale (Cabral et al., 2017; Szakály et al., 2018; Verain et al., 2021). To enable consistency of response between different measures included in the questionnaire and limit participants fatigue, responses were on a 5-point Likert scale ranging from 1 = ‘Not at all important’ to 5 = ‘Extremely important’. The nine factors and the items contained therein, were administered in the standardised sequence originally employed by Steptoe et al. (1995): health; weight control; natural content; mood; sensory appeal; convenience; price; familiarity; ethical concern. Reliability was good for all nine factors with Cronbach’s ranging from α = 0.80 to α = 0.91.

#### Health locus of control

Health locus of control was measured by means of six items derived from the Revised Health Hardiness Inventory (RHHI-24) (Gebhardt et al., 2001). The RHHI-24 was derived from the multi-dimensional health locus of control scale (Wallston et al., 1978), which has been widely validated against dietary outcomes in different societal groups (Cheng et al., 2016). Responses were on a 5-point Likert scale ranging from 1 = ‘Completely disagree’ to 5 = ‘Completely agree’. Given the research focus upon HLoC, the first three items of the IHLoC and EHLoC scales were selected. Items selected to measure IHLoC were: ‘I can be as healthy as I want to be’; ‘I am in control of my health’; ‘I can pretty much stay healthy by taking care of myself’. Items used to measure external HLoC were: ‘I am bored by all the attention that is paid to health and disease prevention’; ‘What’s the use of concerning yourself about your health you’ll only worry yourself to death’; ‘Efforts to improve your health are a waste of time’. Reliability was satisfactory with Cronbach’s α = 0.76 for IHLoC and α = 0.77 for EHLoC.

#### Nutrition self-efficacy

Nutrition self-efficacy (NS-E) was measured using Schwarzer and Renner’s (2000) Perceived Self-Efficacy Scale (PS-ES) which has been widely employed against nutrition outcomes (e.g. Stewart-Knox et al., 2021). The scale was adapted from a 4-point to a 5-point scale to align responses with others in the questionnaire. Respondents were asked how certain they were they could ‘manage to stick to healthy foods, even if… ..’ on a scale ranging from 1 = ‘Very uncertain’ to 5 = ‘Very certain’, in response to the following items: ‘I need a long time to develop the necessary routines’; ‘I have to try several times until it works’; ‘I have to rethink my entire way of nutrition’; ‘I do not receive a great deal of support from others when making my first attempts’; ‘I have to make a detailed plan’. Reliability was good with Cronbach’s α = 0.87. Reliability was very good with Cronbach’s α = 0.90.

### Data analysis

Descriptive statistics consisted of frequencies (*n*; %) or means and standard deviations (SD). Normality of quantitative variables was assessed using skewness and kurtosis. MANCOVA was employed to study associations between food choice motives nutrition self-efficacy and health locus of control whilst controlling for socio-demographic factors and for which post-hoc tests were performed using Sidak’s correction. Separate MANCOVA models were produced for each of the nine food choice motives (Health, Mood, Convenience, Sensory Appeal, Natural Content, Price, Weight Control, Familiarity, Ethical Concern) which were taken as explanatory variables. Nutrition Self-Efficacy (NS-E), Internal Health Locus of Control (IHLoC) and External Health Locus of Control (EHLoC) were entered as covariates, and Country (Germany, Greece, Ireland, Netherlands, Norway, Poland, Portugal, Spain, United Kingdom), gender (man/woman), age group (18–29/30–39/40–54/55–65 years) and education level (low/middle/high) were entered as fixed factors. The null hypothesis was rejected when *p* < 0.05. Effect sizes were estimated using partial eta squared (η^2^_p_). Statistical analysis was conducted using IBM SPSS Statistics, version 28.0 for Windows.

## Results

### Sample description

The sample comprised a total of 9381 participants (49.4% females) recruited in nine European countries: Germany, Greece, Ireland, Netherlands, Norway, Poland, Portugal, Spain and UK (1022–1148 participants from each country). Participants were aged 18–65 years (18–29: *n* = 2063; 30–39: *n* = 2195; 40–54: *n* = 3266; 55–65: *n* = 1857) with similar distribution in three levels of education (low: *n* = 2692; middle: *n* = 3645; high: *n* = 3044) (Table 1). The gender breakdown was fairly similar between countries, however, there were apparent differences in the spread of age and education. Greece was the ‘youngest’ country with 25% falling into the 18–29-year-old category, while Norway was the ‘oldest’ with 27% in the 55–65-year-old age group. There was considerable variation between countries in Education level. Ireland had the greatest proportion of people educated to the highest level, with more than half (50.5%) having obtained a university degree or above. While nearly half (49%) of UK respondents were educated to the lowest level (49%), with relatively few (11%) at the mid-level (11%), Poland had relatively few at the lowest level with more than half (61%) educated to the mid-level.

**Table 1.** Sample characteristics by country (*N* = 9381).

	Norway *n* = 1022	Germany *n* = 1020	Spain *n* = 1025	Greece *n* = 1020	Poland *n* = 1045	UK *n* = 1061	Ireland *n* = 1020	Netherlands *n* = 1020	Portugal *n* = 1148
Gender %									
Men/women	53/47	50/50	51/49	49/51	52/48	51/49	50/50	50/50	50/50
Age group %									
18–29 years	20	19	19	25	24	23	24	20	24
30–39 years	22	16	27	32	24	19	26	18	26
40–54 years	31	41	35	38	28	36	32	38	35
55–65 years	27	24	19	06	24	22	18	24	15
Education %									
Lower	39	30	32	32	11	49	12	29	25
Middle	31	53	43	35	61	15	38	35	38
Higher	30	17	25	33	28	36	50	36	37

UK: United Kingdom.

### Descriptive analyses

The highest mean ranked FCQ factor was Price with Sensory Appeal ranked second and Natural Content third. The lowest ranked FCQ factor was Familiarity (Table 2). Greece, which represented the ‘youngest’ sample across the nine countries, scored higher than any other country on various motives for food choice (health, price, natural content and ethical concern). In contrast, Norway, representing the ‘oldest’ country sample, scored lowest on natural content, weight control and price. The UK sample scored lowest on health as a motivation for food choice.

**Table 2.** Associations between Food Choice Questionnaire factors, nutrition self-efficacy and health related locus of control.

		Nutrition self-efficacy	Internal HLoC	External HLoC
	Mean (SD)	3.37 (0.80)	3.64 (0.78)	2.07 (0.83)
		*r* (*p*)	*r* (*p*)	*r* (*p*)
Health	3.49 (0.74)	0.449 (<0.001)	0.256 (<0.001)	−0.254 (<0.001)
Mood	3.36 (0.83)	0.296 (<0.001)	0.137 (<0.001)	−0.152 (<0.001)
Convenience	3.44 (0.84)	0.051 (<0.001)	0.074 (<0.001)	−0.002 (0.876)
Sensory appeal	3.67 (0.71)	0.144 (<0.001)	0.122 (<0.001)	−0.124 (<0.001)
Natural content	3.57 (0.96)	0.318 (<0.001)	0.142 (<0.001)	−0.225 (<0.001)
Price	3.72 (0.82)	0.057 (<0.001)	0.027 (0.010)	−0.070 (<0.001)
Weight control	3.18 (0.99)	0.278 (<0.001)	0.077 (<0.001)	−0.185 (<0.001)
Familiarity	2.85 (0.89)	0.117 (<0.001)	0.075 (<0.001)	0.046 (<0.001)
Ethical concern	2.91 (1.01)	0.205 (<0.001)	0.075 (<0.001)	−0.057 (<0.001)

*N* = 9381.

*r*: Pearson’s correlation coefficient; HLoC: health locus of control.

The correlations between nutrition self-efficacy (NS-E), internal health locus of control (IHLoC) and external health locus of control (EHLoC) with each food choice motive are presented in Table 2. Higher NS-E and higher IHLoC were associated with higher scores on all food choice questionnaire (FCQ) factors (*p* < 0.001 for all associations). Higher EHLoC was associated with lower scores on the Health, Mood, Sensory Appeal, Natural Content, Price, Weight Control and Ethical Concern factors (*p* < 0.001), and with higher scores on the Familiarity FCQ factor (*p* < 0.001). There was no significant association between EHLoC and the convenience FCQ factor.

### Associations between motives for food choice, nutrition self efficacy and health locus of control

#### Health

The strongest effect size was for the model of Health as a motive for food choice explaining 28.8% of the variance in scores (Table 3). Higher scores on Health were associated with higher Nutrition Self-Efficacy (NS-E), higher Internal HLoC and with lower External HLoC. Scores on the Health factor were higher among participants residing in Germany, Greece and Poland, females and in the middle age groups (30–39 and 40–54 years).

**Table 3.** Relationships of nutrition self-efficacy and health related locus of control with food choice determinants.

	*n*	Multivariate tests		Tests of between-subjects effects															
				Health				Mood				Convenience				Sensory appeal			
		*p*	η^2^_p_	EMM	β	*p*	η^2^_p_	EMM	β	*p*	η^2^_p_	EMM	β	*p*	η^2^_p_	EMM	β	*p*	η^2^_p_
Corrected model	9381					<0.001	0.289			<0.001	0.168			<0.001	0.067			<0.001	0.098
Country		<0.001	0.040			<0.001	0.043			<0.001	0.066			<0.001	0.033			<0.001	0.037
Germany	1020			3.68^f^	0.318	<0.001	0.014	3.39^bcd^	0.284	<0.001	0.007	3.56efg	0.363	<0.001	0.011	3.84^ef^	0.248	<0.001	0.007
Greece	1020			3.66^f^	0.302	<0.001	0.012	3.72^e^	0.613	<0.001	0.034	3.61^efgh^	0.415	<0.001	0.014	3.78^def^	0.183	<0.001	0.004
Ireland	1020			3.45^cde^	0.093	0.001	0.001	3.29^bc^	0.177	<0.001	0.003	3.33^bcd^	0.132	<0.001	0.001	3.53^abc^	−0.067	0.028	0.001
Netherlands	1020			3.31^ab^	−0.052	0.061	0.000	3.16^a^	0.050	0.139	0.000	3.29^abc^	0.092	0.010	0.001	3.45^ab^	−0.142	<0.001	0.002
Norway	1022			3.36^abc^	−0.003	0.912	0.000	3.13^a^	0.022	0.518	0.000	3.43^cde^	0.231	<0.001	0.004	3.53^abc^	−0.060	0.042	0.000
Poland	1045			3.65^f^	0.289	<0.001	0.011	3.67^e^	0.561	<0.001	0.028	3.70^gh^	0.502	<0.001	0.020	3.70^de^	0.104	0.001	0.001
Portugal	1148			3.52^de^	0.155	<0.001	0.003	3.42^cd^	0.310	<0.001	0.009	3.38^bcd^	0.185	<0.001	0.003	3.82^ef^	0.227	<0.001	0.006
Spain	1025			3.41^bcd^	0.046	0.102	0.000	3.39^bcd^	0.276	<0.001	0.007	3.50^defg^	0.301	<0.001	0.007	3.76^def^	0.163	<0.001	0.003
UK (Ref.)	1061			3.36^abc^				3.11^a^				3.20^ab^				3.59^bc^			
Sex		<0.001	0.053			<0.001	0.009			<0.001	0.006			<0.001	0.023			<0.001	0.024
Female	4630			3.55	0.121	<0.001	0.009	3.42	0.115	<0.001	0.006	3.57	0.250	<0.001	0.023	3.77	0.213	<0.001	0.024
Male (Ref.)	4751			3.43				3.31				3.32				3.56			
Age		<0.001	0.022			<0.001	0.007			0.151	0.001			<0.001	0.005			0.002	0.002
18–29 years (Ref.)	2063			3.41^a^				3.39				3.49^bc^				3.63^ab^			
30–39 years	2195			3.46^b^	0.051	0.008	0.001	3.37	−0.019	0.419	0.000	3.51^c^	0.014	0.576	0.000	3.69^c^	0.059	0.005	0.001
40–54 years	3266			3.50^b^	0.091	<0.001	0.003	3.35	−0.045	0.037	0.000	3.44b	−0.048	0.039	0.000	3.70^c^	0.068	<0.001	0.001
55–65 years	1857			3.57^c^	0.160	<0.001	0.007	3.35	−0.043	0.081	0.000	3.34a	−0.155	<0.001	0.004	3.65b^c^	0.026	0.239	0.000
Education		<0.001	0.013			0.139	0.000			<0.001	0.003			0.030	0.001			<0.001	0.002
Low	2692			3.51	0.028	0.102	0.000	3.42^b^	0.101	<0.001	0.003	3.48^a^	0.051	0.020	0.001	3.72^b^	0.081	<0.001	0.002
Middle	3645			3.48	−0.003	0.857	0.000	3.36^a^	0.045	0.018	0.001	3.43^a^	0.001	0.969	0.000	3.65^a^	0.014	0.394	0.000
High (Ref.)	3044			3.48				3.32^a^				3.43^a^				3.63^a^			
Nutrition self-efficacy	9381	<0.001	0.181		0.357	<0.001	0.152		0.266	<0.001	0.063		0.032	0.005	0.001		0.090	<0.001	0.010
Internal HLoC	9381	<0.001	0.028		0.122	<0.001	0.020		0.069	<0.001	0.004		0.073	<0.001	0.004		0.086	<0.001	0.009
External HLoC	9381	<0.001	0.075		−0.125	<0.001	0.024		−0.056	<0.001	0.003		0.038	<0.001	0.001		−0.044	<0.001	0.003
Adjusted *R*^2^				0.288				0.166				0.066				0.097			

	Tests of between-subjects effects																			
	Natural content				Price				Weight control				Familiarity				Ethical concern			
	EMM	β	*p*	η^2^_p_	EMM	β	*p*	η^2^_p_	EMM	β	*p*	η^2^_p_	EMM	β	*p*	η^2^_p_	EMM	β	*p*	η^2^_p_
Corrected model			<0.001	0.250			<0.001	0.116			<0.001	0.187			<0.001	0.120			<0.001	0.135
Country			<0.001	0.109			<0.001	0.091			<0.001	0.078			<0.001	0.086			<0.001	0.088
Germany	3.80^efg^	0.506	<0.001	0.019	3.81^c^	0.327	<0.001	0.009	3.23^def^	0.185	<0.001	0.002	2.88^bcd^	0.318	<0.001	0.008	3.10^de^	0.460	<0.001	0.013
Greece	4.00^gh^	0.712	<0.001	0.038	4.02^d^	0.527	<0.001	0.024	3.51^gh^	0.464	<0.001	0.014	3.00^cde^	0.432	<0.001	0.014	3.36^f^	0.723	<0.001	0.030
Ireland	3.39^c^	0.097	0.009	0.001	3.58^b^	0.091	0.009	0.001	3.15^cde^	0.105	0.008	0.001	2.78^bc^	0.215	<0.001	0.003	2.93^cd^	0.291	<0.001	0.005
Netherlands	3.14^ab^	−0.155	<0.001	0.002	3.55^b^	0.066	0.053	0.000	2.82^b^	−0.228	<0.001	0.004	2.56^a^	0.000	0.991	0.000	2.36^a^	−0.274	<0.001	0.005
Norway	3.23^ab^	−0.057	0.117	0.000	3.22^a^	−0.264	<0.001	0.006	2.69^a^	−0.359	<0.001	0.009	2.47^a^	−0.092	0.012	0.001	2.56^b^	−0.080	0.052	0.000
Poland	3.91^fgh^	0.623	<0.001	0.029	3.87^c^	0.380	<0.001	0.013	3.41^fgh^	0.362	<0.001	0.009	3.30^f^	0.735	<0.001	0.039	3.12^de^	0.480	<0.001	0.013
Portugal	3.77^ef^	0.480	<0.001	0.018	4.02^d^	0.534	<0.001	0.026	3.46^gh^	0.414	<0.001	0.012	3.09^de^	0.529	<0.001	0.022	3.13^de^	0.493	<0.001	0.015
Spain	3.57^d^	0.274	<0.001	0.006	3.86^c^	0.373	<0.001	0.012	3.33^efg^	0.281	<0.001	0.005	3.08^de^	0.512	<0.001	0.020	3.00^cde^	0.367	<0.001	0.008
UK (Ref.)	3.29^bc^				3.49^b^				3.05^cd^				2.56^a^				2.64^b^			
Sex			<0.001	0.009			<0.001	0.009			<0.001	0.025			<0.001	0.003			0.046	0.000
Female	3.65	0.160	<0.001	0.009	3.79	0.147	<0.001	0.009	3.33	0.287	<0.001	0.025	2.90	0.086	<0.001	0.003	2.93	0.039	0.046	0.000
Male (Ref.)	3.49				3.64				3.04				2.81				2.89			
Age			<0.001	0.035			0.001	0.002			<0.001	0.008			0.001	0.002			<0.001	0.009
18–29 years (Ref.)	3.32^a^				3.73^b^				3.07^a^				2.82^a^				2.76^a^			
30–39 years	3.51^b^	0.192	<0.001	0.006	3.73^b^	0.003	0.902	0.000	3.13^a^	0.060	0.029	0.001	2.88^ab^	0.065	0.012	0.001	2.91^b^	0.150	<0.001	0.003
40–54 years	3.65^c^	0.331	<0.001	0.021	3.75^b^	0.019	0.381	0.000	3.21^b^	0.137	<0.001	0.003	2.82^a^	0.004	0.881	0.000	2.95^b^	0.194	<0.001	0.006
55–65 years	3.78^d^	0.460	<0.001	0.030	3.65^a^	−0.074	0.003	0.001	3.32^c^	0.243	<0.001	0.007	2.90^b^	0.085	0.002	0.001	3.03^c^	0.275	<0.001	0.009
Education			0.014	0.001			<0.001	0.008			0.003	0.001			<0.001	0.015			<0.001	0.005
Low	3.59^b^	0.020	0.379	0.000	3.80^c^	0.179	<0.001	0.008	3.23^b^	0.075	0.002	0.001	2.99^c^	0.267	<0.001	0.014	3.01^b^	0.151	<0.001	0.004
Middle	3.53^a^	−0.042	0.046	0.000	3.72^b^	0.093	<0.001	0.002	3.16^a^	0.005	0.841	0.000	2.85^b^	0.126	<0.001	0.004	2.87^a^	0.008	0.726	0.000
High (Ref.)	3.57^ab^				3.62^a^				3.16^a^				2.73^a^				2.86^a^			
Nutrition self-efficacy		0.334	<0.001	0.082		0.032	0.003	0.001		0.302	<0.001	0.059		0.126	<0.001	0.012		0.265	<0.001	0.041
Internal HLoC		0.086	<0.001	0.006		0.044	<0.001	0.002		0.025	0.046	0.000		0.083	<0.001	0.005		0.035	0.009	0.001
External HLoC		−0.112	<0.001	0.011		0.000	0.998	0.000		−0.073	<0.001	0.004		0.137	<.001	0.016		0.055	<0.001	0.002
Adjusted *R*^2^	0.249				0.114				0.186				0.118				0.133			

Letters indicate homogeneous subsets (Sidak’s correction).

EMM: estimated marginal means; HLoC: health locus of control.

#### Mood

The model explained 16.6% of the variance in Mood. Higher scores on Mood were associated with higher NS-E, higher Internal HLoC and lower External HLoC (Table 3). Scores on the Mood factor were highest in Greece and Poland, among those in the lower education level and higher in females than males. Mood scores did not vary by age group.

#### Convenience

The weakest model was for Convenience which explained 6.6% of the variance in scores. Higher scores on Convenience were associated with higher NS-E, higher Internal HLoC and higher External HLoC (Table 3). Convenience was rated more important by those in Poland, which along with Germany, Greece and Spain formed a homogenous subset. Convenience was rated highest by females, those in the 30–39-year-old group and those in the lower educational level.

#### Sensory appeal

The model explained 10% of the variance in Sensory Appeal. Higher scores on Sensory Appeal were associated with higher NS-E, higher Internal HLoC and lower External HLoC (Table 3). Sensory Appeal was rated significantly more important by those residing in Germany, Greece, Portugal and Spain and less important by those in Ireland, Netherlands and Norway. Sensory Appeal was rated significantly higher by females, those in the middle age groups (30–39 and 40–54 years) and those in the lower education level.

#### Natural content

The second largest effect size was for the model of Natural Content which explained 24.9% of the variance. Higher scores on Natural Content were associated with higher NS-E, higher Internal HLoC and lower External HLoC (Table 3). Natural Content was rated more important by those residing in Greece and Germany and least important in Ireland. Natural Content was rated higher by females, those in the middle and higher education levels and with increasing age group.

#### Price

The model explained 11.4% of the variance in Price. Higher scores on Price were associated with higher NS-E and higher Internal HLoC (Table 3). There was no association between Price and External HLoC. Price was rated more important by females and less important by those in the oldest (55–65 years) age group. Price was rated less important with increasing education level.

#### Weight control

The model explained 18.6% of the variance in Weight Control. Higher scores on Weight Control were strongly associated with higher NS-E but only marginally (*p* = 0.046) (given the large sample size and Sidak correction) with higher Internal HLoC. Higher ratings of the importance of Weight Control were also associated with lower External HLoC (Table 3). Greece, Poland and Portugal formed a homogenous group of countries that rated Weight Control higher in importance. Weight Control was rated higher by females, by those in the lower education level and with increasing age group.

#### Familiarity

The model explained 11.8% of variance in Familiarity. Higher scores on Familiarity were associated with higher NS-E, higher Internal HLoC and higher External HLoC (Table 3). Familiarity was rated more important in Greece, Portugal and Spain. Familiarity was rated higher by females, those in the oldest age group (55–65 years) and those at the lower education level.

#### Ethical concern

The model explained 13.3% of variance in Ethical Concern. Higher scores on Ethical Concern were associated with higher NS-E, higher Internal HLoC and higher External HLoC (Table 3). Ethical Concern was rated as more important by those in Greece which together with Germany, Poland, Portugal and Spain comprised a homogenous subset of countries exhibiting higher ratings. Scores on Ethical Concern were higher in females, in those in the lower education level and with increased age.

## Discussion

This secondary analysis has sought to determine and understand potential relationships between food choice motives and nutritional self-efficacy (NS-E), internal health locus of control (HLoC) and external HLoC. Previous research has consistently indicated that taste/sensory appeal is the most important motive for food choice followed by price, health and convenience (Dana et al., 2021; Verain et al., 2022). In contrast, the model showing the strongest effects size indicated by this analysis was for the food choice motive health, 29% of which was explained by NSE, internal and external HLoC. Health and natural content would therefore appear to be the motives most closely associated with NS-E and HLoC. That health was rated lowest of the food choice motives among those in the UK could indicate a greater need to intervene on self-efficacy and locus of control in promoting health eating. The models showing the weakest effect sizes were for convenience and sensory appeal and for which behaviour change factors explained only 7% and 10% of variance respectively implying that NS-E and HLoC are of less relevance to these motives.

As predicted, greater NS-E was associated with greater perceived importance for all food choice motives. That higher NS-E was associated with perceived importance of health as a motive for food choice (which was the strongest model) agrees with other research that found higher NSE was related to healthier food choices (e.g. Appleton and Adams, 2023; Greiner et al., 2018; Kushida et al., 2017; Lo et al., 2019; Smith et al., 2020). Higher NS-E was also associated with higher perceived importance of the weight control motive, which is consistent with previous research indicating that those higher on NS-E are more likely to lose weight through weight loss diets (Annesi, 2015a, 2015b; Freedman and Rubinstein, 2010; Paxton and Sculthorpe, 1999). Together this could imply that NS-E should be taken into account in personalising nutrition intervention to motivate healthy eating and weight loss. In keeping with previous research (Chang et al., 2008; Kavanagh and Bower, 1985; McArthur and Pawlak, 2011), self-efficacy was positively associated with mood, implying that self-efficacy and mood may need to be promoted together in encouraging healthy eating. Further research is needed to understand the nature of the relationship between NS-E and other food choice motives (sensory appeal; convenience; price; familiarity; natural content; ethical concern). Meanwhile, these findings imply that NS-E is a contributing factor in the association between food choice motives and food choices observed in previous research (Alles et al., 2017).

Also as expected, greater internal HLoC was associated with increasing perceived importance in eight out of the nine food choice motives. This corroborates previous studies that have identified links between internal HLoC and healthy eating (e.g. Cheng et al., 2016; Cobb-Clark et al., 2014; Cohen and Azaiza, 2007; Grotz et al., 2011). That natural content, which was the model with the second strongest effect size, was related to higher internal HLoC, is consistent with existing evidence that internal HLoC is related to willingness to pay for organic food (Lee et al., 2019). In keeping with the finding that internal HLoC was associated with ethical concern, internal HlOC has been previously shown to be related to selection of locally produced food (Hempel and Roosen, 2022). Although internal HLoC was only weakly related to the weight control motivation, this corroborates previous research implying that internal HLoC is associated with weight control as a motive for food choice (e.g. Anastasiou et al., 2015) and could have implications for personalising weight control interventions that afford the individual a greater sense of control.

Greater external HLoC was associated with lower perceived importance of the food choice motives health, natural content, weight control, mood and sensory appeal and with greater perceived importance of ethical concern, familiarity and convenience. Relationships between external HLoC and food choice motives were not all in the expected direction. The finding that greater perceived importance of the health motive was associated with lower external HLoC is inconsistent with previous research linking an external HLoC to more risky dietary behaviour (Grotz et al., 2011). Also contrary to expectation, given the price is an external determinant of food choice (and unsurprisingly was rated as more important by those on lower incomes), External HLoC was not associated with price as a motive for food choice. The discrepant findings related to External HLoC could be a consequence of respondents signing up to participate in a study on personalised nutrition via the social research company and which unwittingly biased the sample towards people who were already motivated to make healthy food choices, and who were more likely to volunteer for a health-related research study irrespective of their external HLoC orientation. It is also possible that the unidimensional measure of external HLoC employed in this survey failed to fully capture the construct of externality (Otto et al., 2011).

Our analyses have explored relations between food choice motives, NS-E and HLoC. Whereas food choice motives are dynamic over time and context, NS-E and HLoC, although modifiable to some extent, are more stable constructs. This implies that N-SE and HLoC could be causative in their relationship with food choice motives. The results, therefore, imply that NS-E and HLoC need to be considered in the development of interventions to motivate people to alter their dietary behaviour and/or to make healthier food choices.

Consistent with previous research (Pearcey and Zhan, 2018), food choice motives varied between socio-demographic groups. Females rated all motives as more important, possibly because women are frequently more involved with food (Castellini et al., 2023). Food choice motives also differed between age groups with older people indicating a greater tendency than younger people to rate natural content, weight control, familiarity and ethical concern as high in importance. This is in keeping with previous research indicating that older people are more concerned about healthy eating (e.g. Naughton et al., 2015b), and implies that these motives should be addressed in health eating interventions targeting older people. Price was rated as a less important food choice motive among those in the older age group. Health and sensory appeal were rated higher by those in the middle age group. Also in keeping with previous research (Konttinen et al., 2013), therefore, price and familiarity were rated more highly by those who had spent less time in education. People with less education also ascribed greater importance to Mood, Convenience, Sensory Appeal, Weight Control and Ethical concern than those who were more educated. Those who spent longer in education were more likely to rate price as less important. This analysis has taken education as a marker of socio-economic position. Together, this implies the importance of including affordable and familiar foods in healthy eating interventions. People who were less educated also rated weight control and convenience as important motives for food choice, while those who had spent longer in education rated natural content as more important and price less important. Also, and as previous research has indicated (Pearcey and Zhan, 2018), the relative importance of food choice factors varied between countries. Health and sensory appeal were both rated highest in Germany. Mood, natural content, weight control and ethical concern were rated highest in Greece. Convenience was rated most important highest by those in in Poland and price and familiarity by people in Portugal. Together this implies that strategies to motivate healthier eating may need to be adjusted to meet the varying food choice priorities of citizens in different countries.

## Conclusion

This research is novel in investigating food choice motives and potential associations with NS-E and HLoC in a large international sample. Although relevant associations were found, the study was cross-sectional and correlational which limits the degree to which conclusions can be drawn on causal relationships between food choice motives and behaviour change factors. We therefore recommend future reserch to use experimental paradigms that can establish causality. Another limitation is that the FCQ and NSE scales, which were originally validated as 4-point scales, were only converted to 5-point scales for the purpose of this study, when it is becoming increasingly recognised that extended Likert scales can be more sensitive, particularly for cross-cultural studies (Ares, 2018; Cunha et al., 2018). The 5-point scales used here, although more sensitive than the original 4-point scales, could have limited the discriminate ability of the measures. The apparent clarity of these results, however, suggests the scales used in this analysis were effective for the purpose of the research. Meanwhile, extended scales are recommended for future research. A further potential limitation is that data were collected some years ago. Given the research is focused upon enduring traits rather topical issues, however, suggests that the this is unlikely to limit ability to draw conclusions. Meanwhile, food choice and relationship to theories of behaviour change such as HloC, remains an under-researched area and worthy of further study (Hempel and Roosen, 2022).

To conclude, whereas associations between food choice motives and internal HLoC were all in the expected direction, associations with external HLoC were less clear and will require further study. That people with higher self-efficacy scored higher on all food choice motives implies that intervention to motivate people on food choice behaviour, would need to take self-efficacy into account. Individuals with a low self-efficacy, low internal and a high external HLoC who want to eat more healthily could be encouraged to take control by looking at food label information, by monitoring and providing feedback on dietary behaviour which can then be adjusted by the person on a continuous basis.
